# Preoperative visualization of the greater occipital nerve with magnetic resonance imaging in candidates for occipital nerve decompression for headaches

**DOI:** 10.1038/s41598-024-65334-4

**Published:** 2024-07-02

**Authors:** Mariam Saad, Isaac V. Manzanera Esteve, Adam G. Evans, Huseyin Karagoz, Tigran Kesayan, Krista Brooks-Horrar, Saikat Sengupta, Ryan Robison, Brian Johnson, Richard Dortch, Wesley P. Thayer, Patrick Assi, Lisa Gfrerer, Salam Kassis

**Affiliations:** 1 Department of Plastic Surgery, Vanderbilt University Medical Center, Nashville, TN 37232 USA; 2 Department of Anesthesiology, Department of Neurology, Vanderbilt University Medical Center, Nashville, TN 37232 USA; 3 Department of Neurology, Tennessee Valley Healthcare System, Nashville, TN 37212 USA; 4 Vanderbilt University Institute of Imaging Science, Vanderbilt University Medical Center, Nashville, TN 37232 USA; 5 Philips Healthcare, Nashville, TN 37219 USA; 6 University of Texas Southwestern Medical Center, Dallas, TX 75390 USA; 7 Barrow Neurological Institute, Phoenix, AZ 85013 USA; 8 Division of Plastic and Reconstructive Surgery, Weill Cornell, New York, NY 10065 USA

**Keywords:** Headache surgery, Decompression surgery, MRI, Occipital nerve, Neuralgia, Neuropathic pain, Migraine surgery, Nerve, Nerve decompression, Peripheral nervous system, Headache, Migraine, Diagnostic markers

## Abstract

Occipital nerve decompression is effective in reducing headache symptoms in select patients with migraine and occipital neuralgia. Eligibility for surgery relies on subjective symptoms and responses to nerve blocks and Onabotulinum toxin A (Botox) injections. No validated objective method exists for detecting occipital headache pathologies. The purpose of the study is to explore the potential of high-resolution Magnetic Resolution Imaging (MRI) in identifying greater occipital nerve (GON) pathologies in chronic headache patients. The MRI protocol included three sequences targeting fat-suppressed fluid-sensitive T2-weighted signals. Visualization of the GON involved generating 2-D image slices with sequential rotation to track the nerve course. Twelve patients underwent pre-surgical MRI assessment. MRI identified four main pathologies that were validated against intra-operative examination: GON entanglement by the occipital artery, increased nerve thickness and hyperintensity suggesting inflammation compared to the non-symptomatic contralateral side, early GON branching with rejoining at a distal point, and a connection between the GON and the lesser occipital nerve. MRI possesses the ability to visualize the GON and identify suspected trigger points associated with headache symptoms. This case series highlights MRI's potential to provide objective evidence of nerve pathology. Further research is warranted to establish MRI as a gold standard for diagnosing extracranial contributors in headaches.

## Introduction

The occipital nerves originating from the C2 and C3 spinal nerves provide sensation to the posterior head, encompassing the area beneath the occiput to the cranial vertex and extending laterally to the ear and the skin above the parotid gland^1^. Compression of the occipital nerves at specific points along their pathways can trigger occipital neuralgia and cervicogenic headache^2^. The relief of symptoms by local anesthetic blockade is a diagnostic criterion in both types of headaches as specified by the International Classification of Headache Disorders 3^rd^ Edition (ICDH-3)^3^. Migraines, however, have traditionally been attributed to have central nervous system origins, with more recent reports proposing a pathogenesis involving both central and peripheral mechanisms, including the role of pain from peripheral nerves of the head^4–7^. The ongoing debate centers on determining the primary site of the pathology within this complex interplay^4^.

Migraine headaches affect approximately 15–18% of the global population^8^, and rank as the second leading cause of disability worldwide^9,10^. In the United States, migraines are the most prevalent condition contributing to lost work hours, constituting a significant financial burden of $19.6 billion annually^11^. Symptomatic management with abortive and preventive medications remains the mainstay of migraine treatment^12^. Migraine prevention is recommended for patients experiencing more than four migraine episodes a month, yet less than half of these patients receive maintenance preventative therapy^13^. The range of medications used to treat migraines spans at least five different drug classes^14^, highlighting the multifaceted nature as well as the need for more definitive treatments for this disease. Individuals with symptoms refractory to medications and experiencing a characteristic headache in a distribution of peripheral nerves along the scalp could be suffering from headaches triggered by an external nerve compression^15–17^. Chronic headaches that meet diagnostic criteria for chronic migraine may also be accompanied by pain in a peripheral nerve distribution, such as the occipital nerve, due to a variety of etiologies. Although there are no reliable studies of how many patients with chronic migraine also have occipital neuralgia diagnosis, some estimates suggest that up to a quarter of patients presenting to a community headache clinic have a pain disorder associated with the occipital nerve^18^.

Furthermore, 85% of patients with occipital nerve area pain who present to a headache clinic are diagnosed with a second headache diagnosis, primarily migraine^18^. Chronic pain of a cranial nerve may lead to allodynia, which is known to lead to chronification of migraine headaches, and patients with painful neuropathy of the occipital nerve who also have chronic migraines may need additional treatment considerations. Patients in this category may find relief through treatments with peripheral nerve cascade effects, such as botulinum toxin injections, nerve blocks, transcutaneous nerve stimulation, implanted nerve stimulators, radiofrequency nerve ablation (RFA), and cryoneurolysis^19–22^. Yet these treatments are recognized for their short-lived temporary effects^23^.

Surgical decompression of the occipital nerves at their origin has been an established treatment for persistent cervicogenic headaches using dorsal C2 root decompression or anterior cervical decompression and fusion^24–26^. Additionally, surgical decompression along the distal tracks of occipital nerves, beyond their spinal origins, has shown significant improvement in patients with occipital neuralgia^27,28^. For migraines, studies have found extracranial nerve decompression surgery to be highly effective in reducing or eliminating symptoms in a subset of patients^29–34^. With careful patient selection, 50 to 85% experience complete symptom resolution, with an additional 8 to 30% report significant improvement^35–38^. Patient candidacy for decompression surgery relies on a comprehensive clinical evaluation, incorporating detailed history, headache characteristics, patient headache sketches, and response to injectables, with a lack of reliance on direct objective measures^37^.

In diseases related to the anatomic compression of peripheral nerves, such as neuropathies of the brachial and lumbosacral plexuses and carpal and cubital tunnel syndromes, specialized imaging modalities have been developed to identify the anatomic landmarks causing the entrapment of peripheral nerves and visualizing the compression points^39–44^. These techniques aid in diagnosis, particularly when clinical evaluation is insufficient or inconclusive and when nerve conduction studies are challenging^45^. In the case of occipital headaches, ultrasound imaging has been described to identify sites of occipital nerve entrapment in occipital neuralgia^46^, yet evaluation with ultrasound relies heavily on operator expertise in performing the test and analyzing the results, making it operator-dependent^2^. In cervicogenic headaches, MRI is used to detect structural changes of ligaments and membranes in the upper cervical spine that are postulated to be the sources of these headaches^47,48^. However, the diagnostic value of these changes is still controversial^49^.

High-resolution MRI can help identify peripheral nerve pathologies by detecting nerve signal abnormalities, recognizing muscle-related changes within a particular nerve region, displaying unexpected lesions that imitate symptoms of nerve damage, or ruling out neuropathy by revealing entirely normal imaging features in both muscles and nerves^50^. To our knowledge, no imaging technique has been identified that can successfully trace the occipital nerves and identify nerve pathologies with possible applicability for migraines and occipital neuralgia.

In this study, we aim to explore the potential of high-resolution MRI in identifying nerve pathologies in the GONs of patients with migraines undergoing occipital nerve decompression surgery. MRI findings are compared to operative observations, and patient-reported outcomes are collected to validate the observed pathologic findings.

## Patients and methods

This study was conducted in accordance with the principles outlined in the Declaration of Helsinki. The study protocol was approved by the Institutional Review Board (IRB) of Vanderbilt University Medical Center. All participants were provided written informed consent before enrollment, and confidentiality of their data was strictly maintained throughout the study duration. Participants were assured of their right to withdraw from the study without repercussion. Individuals scheduled to undergo occipital nerve decompression surgery were approached for potential participation. The inclusion criteria encompassed males and females aged 18–85 who were capable of undergoing MRI scanning, with a diagnosis of chronic headache in the occipital region, and who could provide consent. Exclusion criteria included patients unable to recline in the MRI due to orthopedic, cardiac, or claustrophobic reasons, as well as children, patients in pregnancy, and individuals with metallic implants or previous exposure to metal fragments.

The occipital trigger site comprises three cranial sensory nerves that can be compressed by surrounding tissue: the GON, the lesser occipital nerve (LON), and the third occipital nerve (TON). The GON is thicker in caliber than the two other nerves and is characterized by radiation of the pain from the back of the head to the apex^37^. The LON is characterized by pain that starts posterolateral in the scalp and that can radiate towards the ear^37^. In this study, patients with headache characteristics congruent with compression of the GON are included.

All patients underwent comprehensive evaluation by a neurologist and a pain medicine physician and were given the ICHD-3 diagnosis of chronic migraine. Furthermore, all patients had pain of varying durations in the area of the occipital nerves, from frequent several-second episodes to constant baseline pain with superimposed episodes of worsening pain. All patients described this occipital pain as a contributor to their migraine headache episodes. Although the pain in some of these participants was not strictly brief and paroxysmal, which is typical for a ‘neuralgia’ diagnosis, their pain was neuralgiform in nature, severe, and they met all other diagnostic criteria for occipital neuralgia per the ICHD-3^3^. The patients were evaluated to distinguish occipital neuralgia from atlantooccipital, atlantoaxial joint, or cervical zygapophyseal joint etiologies. All patients had failed medical therapy and demonstrated temporary relief with nerve blocks or trigger point injections. Subsequently, patients were referred to the Department of Plastic Surgery and were deemed appropriate candidates for decompression surgery. Patients presented for MRI imaging of the occipital region on average three days prior to the scheduled surgery. All imaging was performed at Vanderbilt University Medical Center. The acquisition and subsequent analysis of all MRIs were conducted by the first author, I.M.E., who remained blinded to any patient-specific symptoms or complaints. All surgeries were performed at the same institution and video-recorded by the senior author, S.K., who described the operative pathologic findings, completely blinded from the MRI results. Additionally, two independent surgeons, H.K. and P.A., reviewed the video recordings to identify pathologic findings, blinded from the MRI results.

Patients were asked to complete a questionnaire via Research Electronic Data Capture (REDCap), a secure data collection tool that meets HIPAA compliance standards, to obtain average migraine pain severity, monthly migraine days, and headache duration when untreated with medication before surgery and at a 6-month post-operative timepoint. The percent change of these three factors between the pre- and post-operative settings was calculated^51^.

### MRI Technique

MRI was performed using a research 3-Tesla Philips Healthcare MRI (Philips North America Corporation, Cambridge, MA, USA) and a 32-channel head coil. The protocol included three MRI sequences characterized by targeting fat-suppressed fluid-sensitive T2-weighted signal intensity to enhance nerve pathologies and perform a morphological assessment (nerve course caliber and size)^50^. The three MRI sequences are T2-FFE (3D PSIF), Fast inversion recovery (3D NerveView) and 3D mDixon TSE (3D BrainView). The sequence parameters are summarized in Table 1.

**Table 1:** Imaging parameters.

Sequences	T2-FFE (3D PSIF)	Fast inversion recovery (3D NerveView)	3D mDixon TSE (3D BrainView_T2)
Field-of-view (mm^3^)	200 × 200 × 250	200 × 200 × 170	220 × 220 × 170
Resolution (mm^3^)	0.89 × 0.89 × 0.89	0.89 × 0.89 × 2	0.89 × 0.89 × 0.89
Slice Orientation	Sagittal	Sagittal	Sagittal
TE/TR (ms)	4.09/8.9	185/2200	216/2000
Scan time (min: sec)	7:32	5: 32	5: 10

*T2-FFE* T2-weighted fast field echo, *PSIF* fast imaging with steady state precession by Siemens, *3D* three dimensions, *TE* echo time, *TR* repetition time.

### MRI analysis

Nerve tracking was achieved using Connectome Workbench version 1.4.2. This tool overlays and reformats the images of the three sequences simultaneously in any plane, including the oblique plane, without loss of resolution, allowing the visualization of nerves with a chosen contrast regardless of location and orientation.

Analysis of the GON topography was performed by following and tracking the nerve distally in the subcutaneous tissue, then proximally at the trapezius muscle and fascia, the splenius capitis muscle, the semispinalis capitis muscle, and the obliquus capitis muscles until reaching the origin of the nerve proximally at the level of the C2 and C3 vertebrae. Diverse aspects such as nerve pathway variations, nerve caliber changes, and interactions between the nerve and adjacent structures were documented to identify pathologies linked to the GON. Such structures include the occipital artery (OA), the LON, and the muscles and fascia coursing along the track of the GON. Additionally, variations in nerve intensity signal at certain locations can be associated with nerve inflammation^52^.

### Surgical technique and outcomes

Surgery is performed using a transverse occipital incision that allows access to the greater, lesser, and third occipital nerves. The LON is exposed laterally and tracked proximally to its exit from the posterior border of the sternocleidomastoid muscle and distally to the subcutaneous tissues, releasing all compression points. The GON is first identified at the third compression point as it exits the semispinalis capitis muscle and is tracked distally through the fourth compression point upon entry into the trapezius muscle fascial canal, followed by the fifth compression point at a potential intersection with the OA. Lastly, the sixth and most distal compression point is located at the trapezius fascia at the level of the nuchal ridge reaching into the subcutaneous tissue. The nerve is then tracked proximally within the semispinalis capitis muscle to the second compression point at the entry into the semispinalis capitis muscle, followed by the first and most proximal compression point at the junction of obliquus capitis inferior and the semispinalis capitis muscles^15,35,53^. The TON is identified caudally to the GON and decompressed as well.

## Results

### Patient characteristics

Twelve patients were recruited to participate in this study. Ten were female and the average age was 49 ± 15. As a baseline, the average frequency of headaches was 16.5 ± 8.9 days/month, the mean headache duration was 20.0 ± 12.5 h/day, and the average pain level was 7.5 ± 1.4 on a scale from 1 to 10. All patients have had a previous trial of at least one injection with temporary relief of headache symptoms, with nerve blocks being the most commonly used (75%). Nausea (83%) and photophobia (83%) were the most prevalent symptoms associated with the headaches. A family history of migraine headaches was present in 58% of patients. All patients have had trials of many medications prior to undergoing surgery. Most notably, all patients have been on tricyclic antidepressants or serotonin-norepinephrine reuptake inhibitor (100%). The majority have used calcitonin-gene related peptide inhibitors (91.7%), topiramate (75%), other anti-epileptic drugs (91.7%), and non-steroidal anti-inflammatory drugs (91.7%). Patient demographics, headache characteristics and, previous treatments are summarized in Table 2.

**Table 2:** Patient demographics, headache characteristics, and previous treatments.

Demographics	Number of patients (n = 12)
Age ± SD	48.5 ± 15.1
Sex, n (%)	
Female	10 (83.3%)
Male	2 (16.7%)
Headache characteristics ± SD	
Headache frequency (days)	16.5 ± 8.9
Headache duration (hour)	20.0 ± 12.5
Headache pain (scale 1–10) ± SD	7.5 ± 1.4
Associated headache symptoms, n (%)	
Nausea	10 (83.3%)
Photophobia	10 (83.3%)
Blurry vision	8 (66.7%)
Difficulty concentrating	5 (41.7%)
Previous injection treatment, n (%)	
Botox	8 (66.7%)
Nerve block	9 (75.0%)
RFA*	4 (33.3%)
History, n (%)	
Family history of migraine	7 (58.3%)
Failed medical therapy	
Beta Blocker	9 (75.0%)
Tricyclic antidepressant (TCA) or Serotonin-norepinephrine reuptake inhibitor (SNRI)	12 (100%)
Topiramate	9 (75.0%)
Other anti-epileptic drug (AED)	11 (91.7%)
Angiotensin-converting enzyme (ACE) inhibitor	4 (33.3%)
Calcitonin-gene related peptide (CGRP) inhibitor	11 (91.7%)
Memantine	5 (41.7%)
Non-steroidal anti-inflammatory drugs (NSAIDs)	11 (91.7%)
Opioids	3 (25.0%)
Acetaminophen	8 (66.7%)

*SD* standard deviation.

*RFA, radiofrequency ablation of the medial branches occurred at the level of C3-C5 joints or the C2-C4 joints.

### MRI

The MRI protocol facilitated the visualization of the GON, covering its entire span from its most distal point in the subcutaneous tissue until its origin at the C2 and C3 vertebrae proximally. The nerve was traced using axial, parasagittal, and coronal slices, and rotated in a manner that enabled the visualization of both longitudinal and transverse sections at any stage of the analysis.

**Figure 1:**
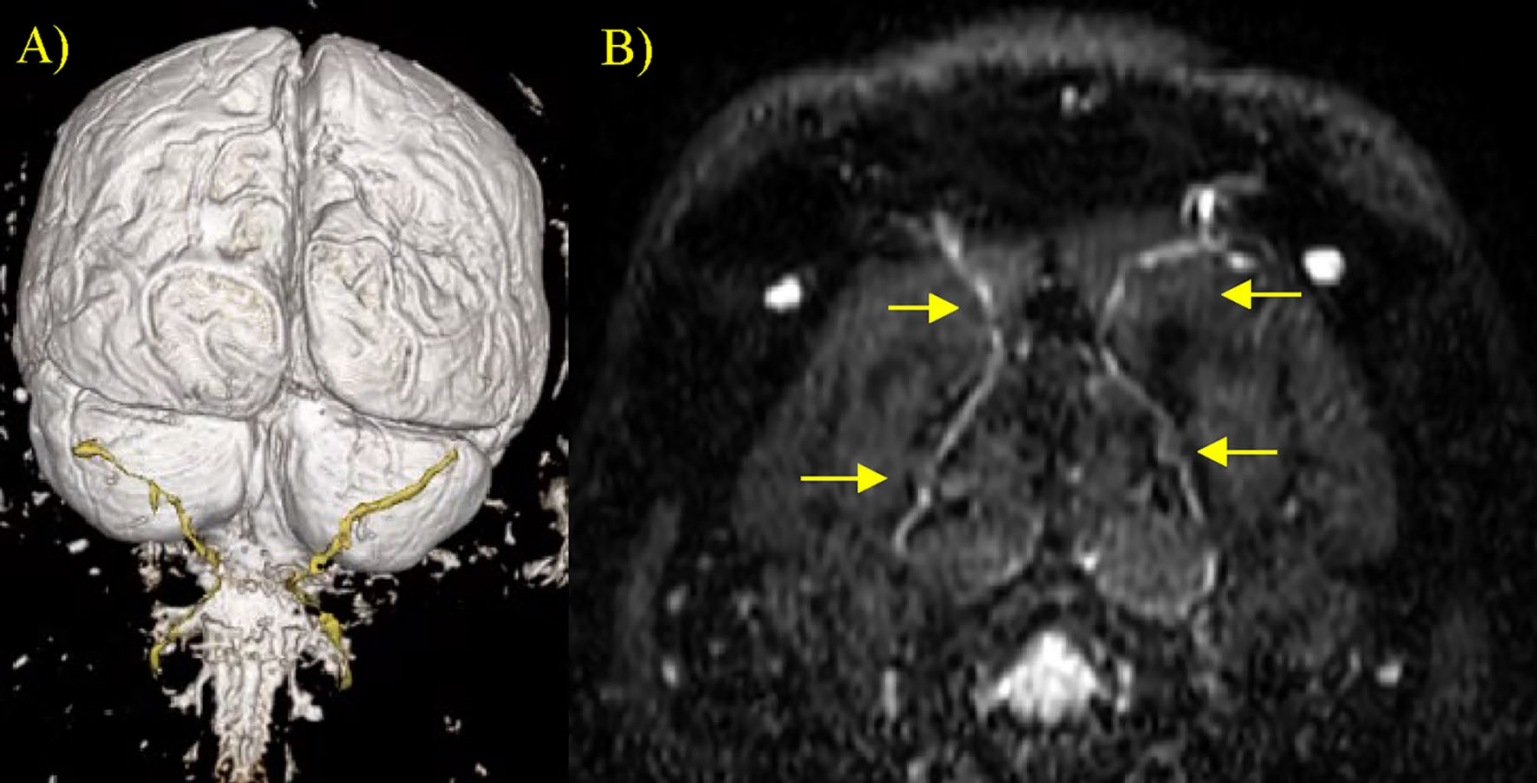
Tracking of the GONs. (**A**) A representative 3D map of the left and right occipital nerves. (**B**) NerveView image of the GON bilaterally near the obliquus capitis muscle up to the trapezius muscle with yellow arrows pointing at the nerve path.

Continued reformatting of the three superimposed MR images in any desired plane while maintaining resolution enables comprehensive visualization of the nerve’s entire pathway. Additionally, the image overlay feature permits the selection of a preferred contrast for enhanced clarity. A representative 3-D visualization of the bilateral GONs’ course is shown in Fig. 1-A of a patient with migraine and associated pain in bilateral areas of the occipital scalp. Performing transverse imaging and capturing both the left and right GONs in view facilitates a comparative analysis between the pathological side and its counterpart, as shown in Fig. 1-B of the same patient.

Among the twelve patients who underwent imaging, four MRI observations were particularly prominent and were also encountered intra-operatively and upon review of the video recordings. These observations are summarized in Table 3.

**Table 3:** Summary table of neuropathies detected on MRI and intraoperatively.

Neuropathy	Number of Patients with neuropathy detected on MRI	Number of Patients with neuropathy detected intraoperatively	Number of Patients with neuropathy detected intraoperatively but not on MRI
Entanglement of the GON by the occipital artery	5	7	2
Abrupt change in GON caliber with high intensity signal	4	6	2
Early branching of the GON	2	3	1
Connection between the GON and the LON	2	2	0
Total Patients	12		

In this paper, we will outline each of the four identified neuropathies on MRI scans that were confirmed by intra-operative examination. Some of the neuropathies were observed in patients intra-operatively but were not detected on pre-operative MRI. It should be emphasized that patients may have more than one of these neuropathies, but our focus will be on describing these individual findings comprehensively.

**Figure 2:**
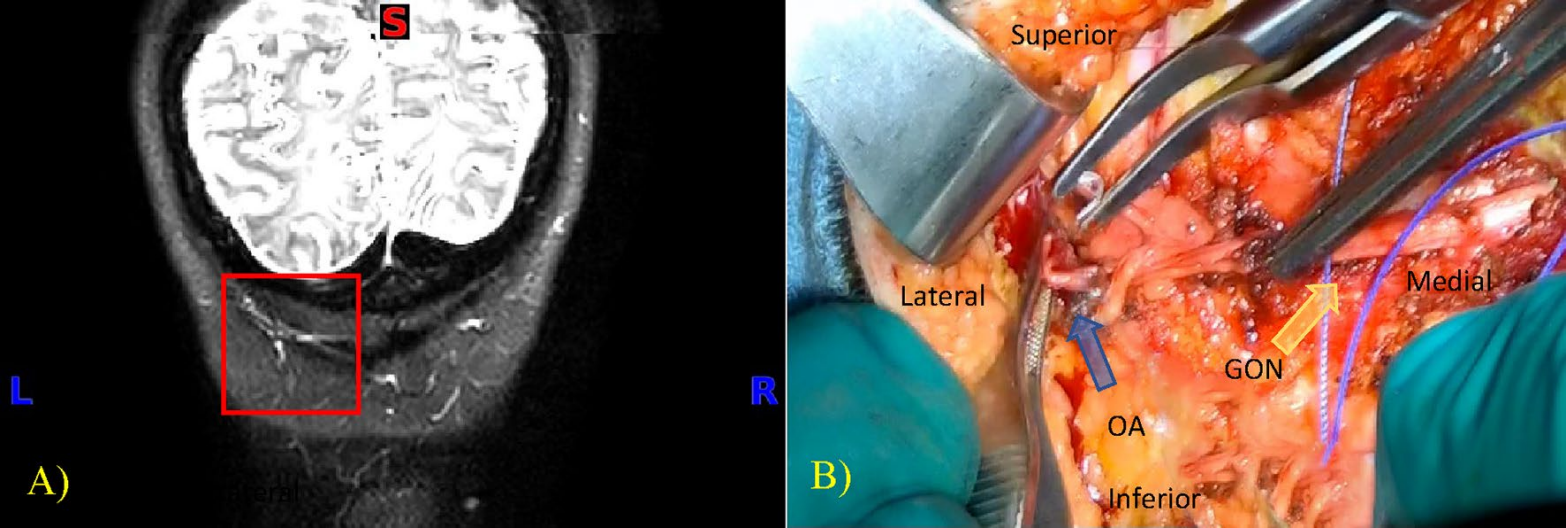
Coronal NerveView MRI tracking the left GON. (**A**) Two crossing structures with high intensity signals (red box). (**B**) Intraoperative image showing the GON (yellow dotted lines) and the OA being clipped and separated from the GON.

The most observed finding on the MRI that translated intra-operatively into a pathological compression point is the entanglement of the GON by the OA. This finding was identified in five patients on the MRI and confirmed in these same five patients intra-operatively, and seen again in additional two patients intra-operatively, yielding a successful detection rate of 71.4% by MRI compared to intra-operative findings. The OA was successfully managed by clipping and transecting the artery and detangling it from around the GON. MRI imaging and an operative photograph of a 51-year-old patient with migraines, predominantly in the left occipital region, depict the first pathology in Fig. 2.

**Figure 3:**
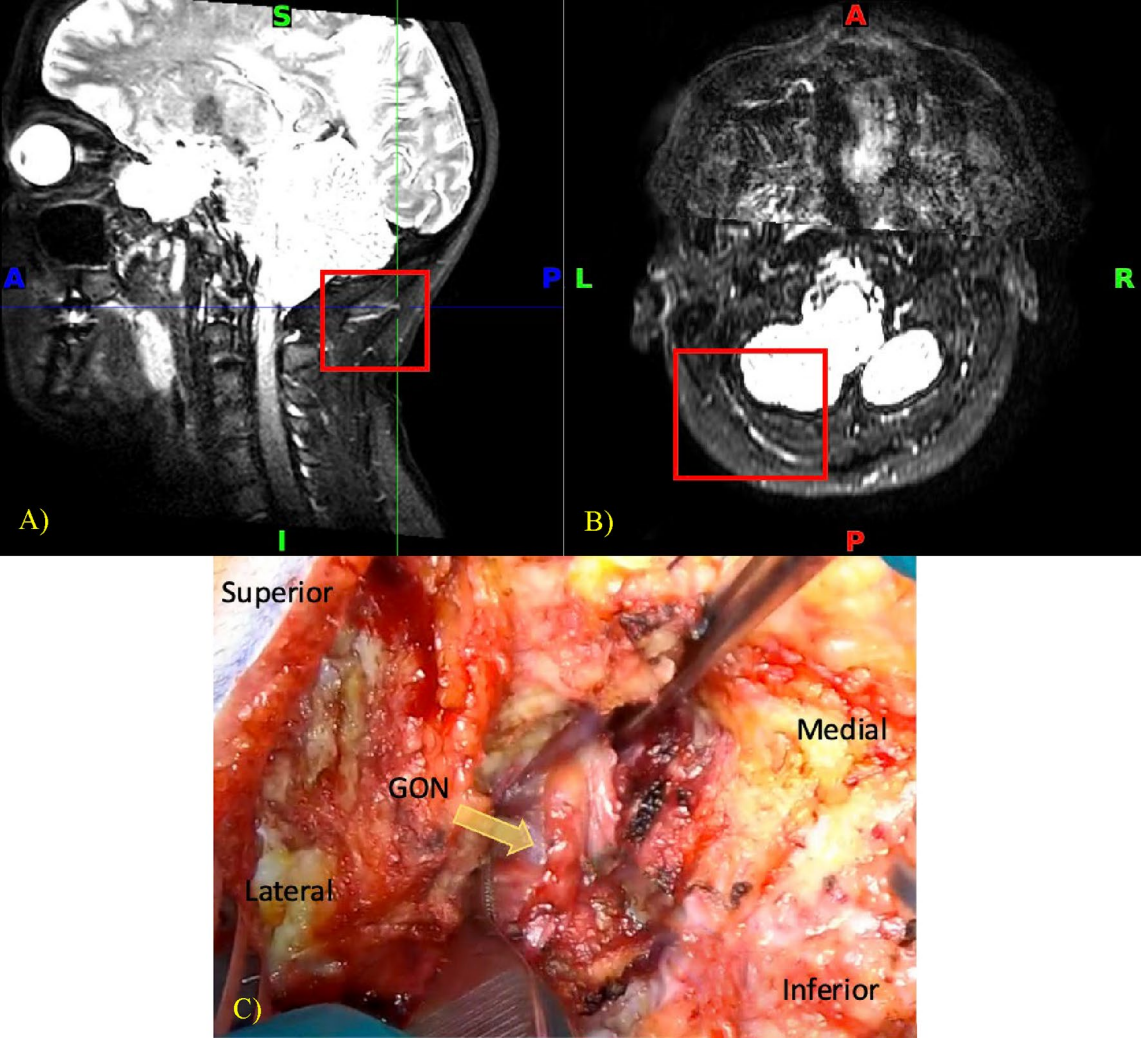
(**A**) Sagittal and (**B**) Transverse NerveView MRI tracking the left GON with high intensity signal as it courses through the trapezius fascia (red box). (**C**) Intraoperative thickened and yellowish GON at the level of a thick trapezius fascia (yellow arrow).

**Figure 4:**
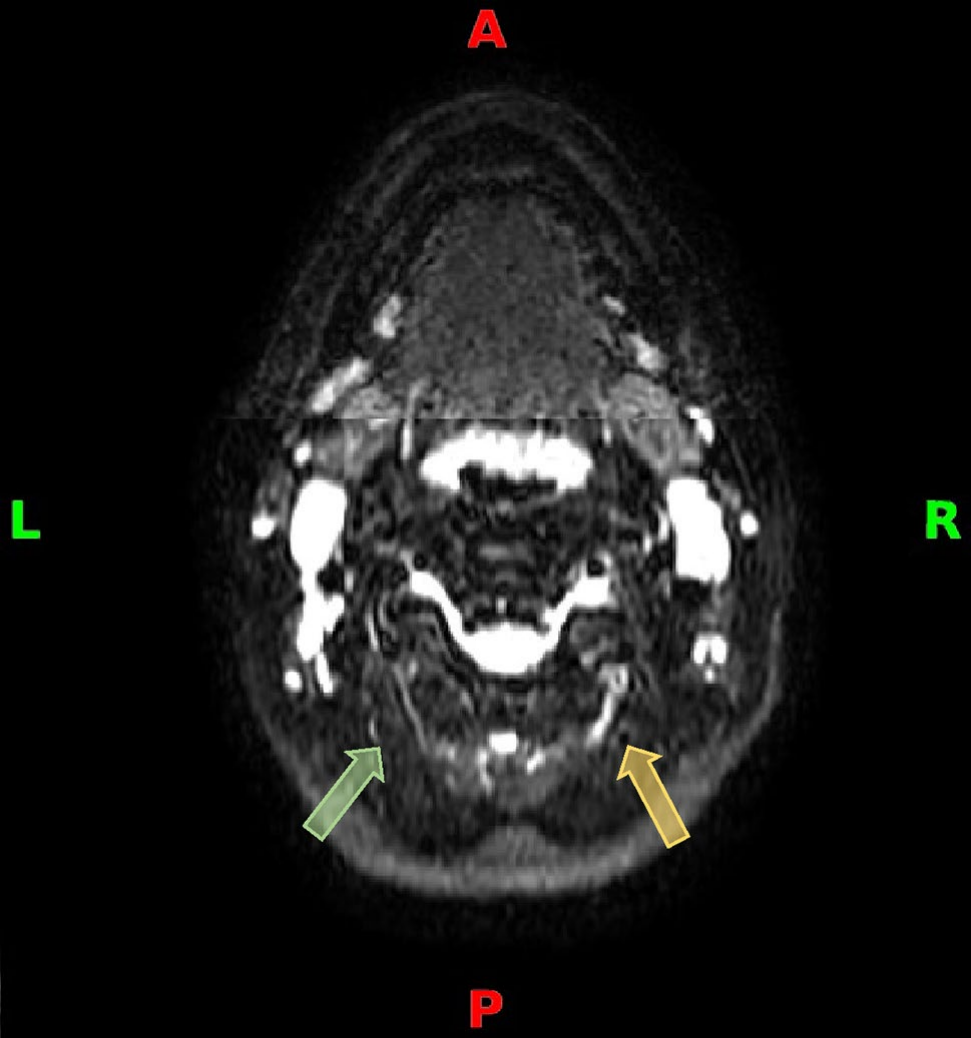
Transverse NerveView MRI tracking the right GON (yellow arrow) with high intensity signal as compared to the left GON (green arrow).

In four patients, sections of the GON at particular locations were identified on the MRI with higher signal intensity and increased thickness. These were interpreted as signs of inflammation (Fig. 3). Operative assessment of the GON and review of the video recording validated the finding as a thickened and edematous nerve with a yellowish discoloration that diminished upon decompression and was classified as inflammation by the surgeons in six patients. This neuropathy was successfully detected by MRI in 66.7% of cases. The findings of high signal intensity and increased thickness are shown in Fig. 3, using images of a 48-year-old patient with symptoms predominantly on the left side. In some cases, the nerve is described intraoperatively as demonstrating an hourglass deformity. An MRI of another patient, who predominantly suffers from right-sided migraines, shows the same pathology in Fig. 4. Yet in this patient, the right occipital nerve exhibits higher signal intensity compared to its counterpart on the left. The intraoperative photo corresponding to the MRI in Fig. 4 is shown in Fig. 6-B, where only the right occipital region is being operated on, and where the patient is demonstrating two types of neuropathies.

**Figure 5:**
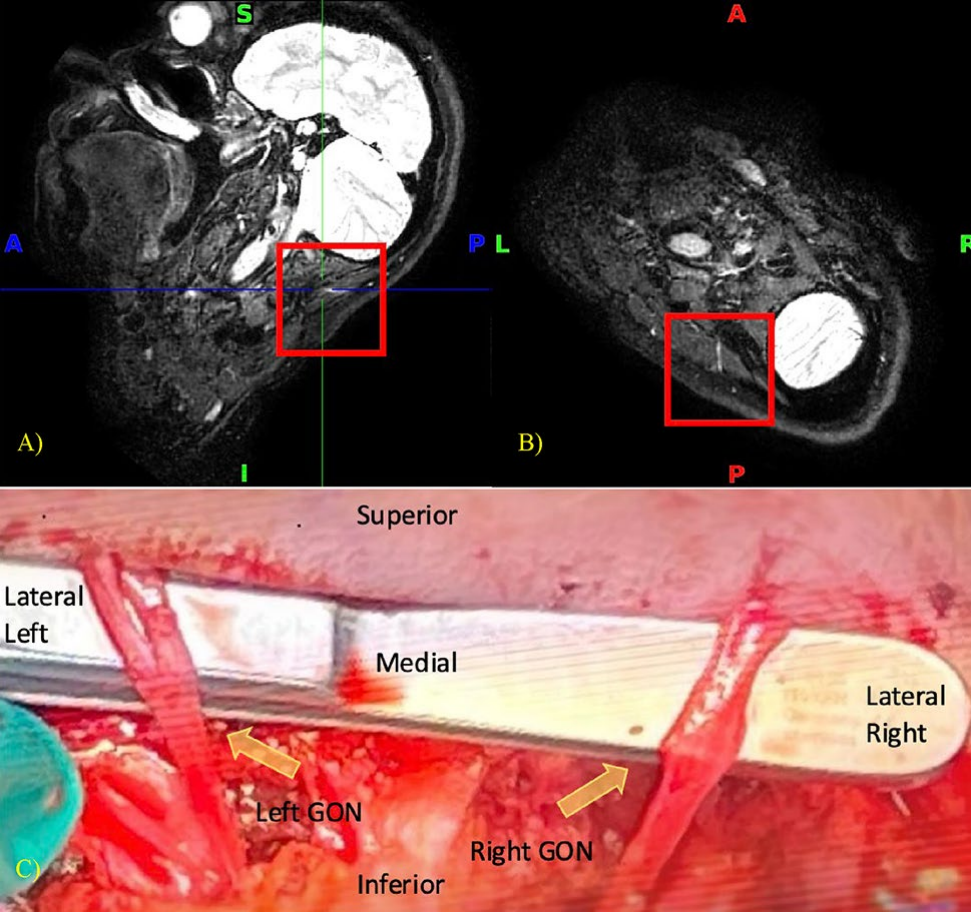
(**A**) Sagittal and (**B**) Transverse BrainView MRI showing a split structure suspected to be the GON (red box). (**C**) Intraoperative image showing the splitting in the left GON (yellow dotted lines) and the absence of splitting in the right GON.

Anatomical variations of the GON can also represent possible points of neuropathy. Some variations can include early branching of the nerve and rejoining at a distal point with structures passing through the splitting. The branching was determined on the MRI and later observed again during surgery in two out of three patients, giving an MRI a detection rate of 66.7% for this neuropathy. Figure 5 demonstrates the early branching in a 53-year-old patient with predominant migraines on the right side.

**Figure 6:**
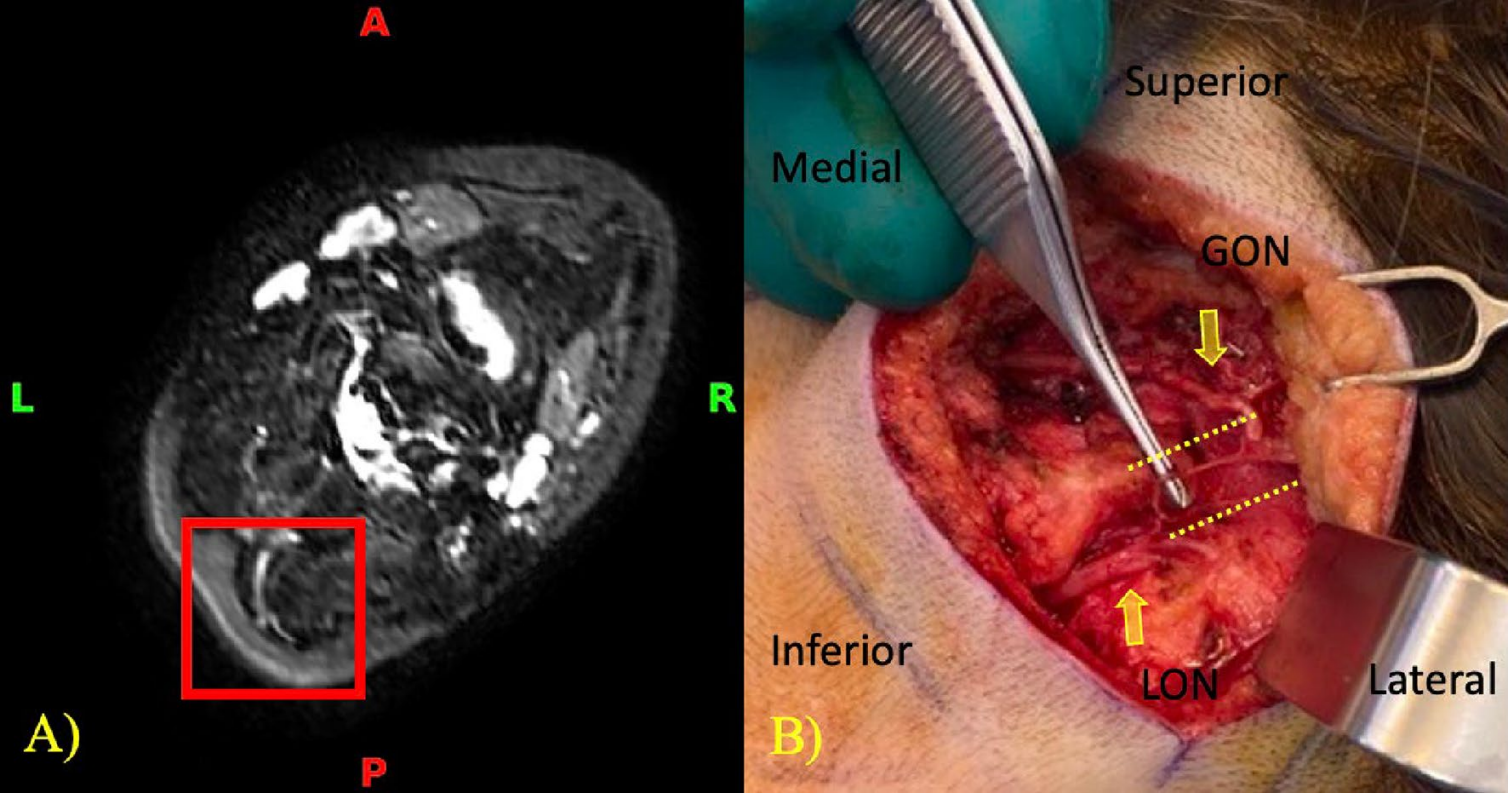
(**A**) Transverse NerveView MRI showing connected structures (red box), appearing to depict the right GON and LON. (**B**) Intraoperative image showing the GON and the LON (yellow arrows) having a neural connection (yellow dotted lines).

The relationship of the GON relative to its surroundings is an important point that was being monitored on MRI. Two patients had images demonstrating a connection between the GON and LON. The relationship was seen again intraoperatively in these same two patients and is depicted in Fig. 6 with a 25-year-old patient having predominant migraines on the right side. The detection rate of the MRI of a connection between GON and LON in this series is 100%. It is important to mention that the MRI was capable of detecting both neuropathies along the course of the right GON in this patient: the high signal intensity signal at the level of the paraspinal muscles shown in Fig. 4 and the connection between the GON and the LON shown in Fig. 6.

### Post-operative outcomes

Responses to the post-operative questionnaire were collected at 6 months and demonstrated a 64.2% decrease in monthly migraine days from an average of 16.5 ± 8.9 days to 5.9 ± 6.8 (*p* < 0.0001). When headaches did occur, their mean duration decreased by 73.5%, from an average of 20.0 ± 12.5 h to a post-operative average of 5.3 ± 7.3 h (*p* < 0.0001). Headache pain scores decreased by 18.7%, from an average of 7.5 ± 1.4 to a post-operative average of 6.1 ± 2.4 (*p* = 0.004). The changes in patient outcomes are summarized in Table 4.

**Table 4:** Patient pre-operative and post-operative headache outcomes.

Occipital (n = 12)	Preoperative	Postoperative	Percent change	Mean improvement	95% CI	*p*-value
Monthly Migraine Days ± SD	16.5 ± 8.9	5.9 ± 6.8	− 64.2%	10.6 ± 1.9	[6.6–14.6]	< 0.0001
Headache duration (hour) ± SD	20.0 ± 12.5	5.3 ± 7.3	− 73.5%	14.7 ± 13.8	[8.4–21.0]	< 0.0001
Headache pain (scale 1–10) ± SD	7.5 ± 1.4	6.1 ± 2.4	− 18.7%	1.4 ± 0.4	[0.5–2.3]	0.004

*SD* standard deviation.

## Discussion

In most patients, the pre-operative MRI was able to visualize and track the GON from its proximal origin at the C2 and C3 vertebrae to the subcutaneous tissue passing through the described six compression points^15,35,53^. In each MRI scan, abrupt changes in nerve thickness, nerve signal intensity, and variable relationships with nearby structures were being evaluated.

Among the twelve patients, several anatomic variations were noted. The OA was observed entangling the GON on the MRI in five patients. The OA compressing the GON is a previously established migraine trigger and compression point that would necessitate intervention to achieve symptomatic improvement^54^. In cadaveric studies, the relationship between the GON and OA appears intricate, as they might run in a parallel fashion at certain junctures and intersect at others. In a study involving 73 cadavers, the GON and OA had crossed once in 78% of cases, twice in 7%, and three times in 4%^55^. Additional research is needed to investigate whether interactions between the OA and the GON are associated with symptomatic improvement following surgery. Another four patients had a hyperintense signal emission on the MRI from their GON, especially when compared to the contralateral nerve in the case of unilateral headaches, hypothesized to represent inflammation. The nerves were seen intra-operatively as thickened, edematous, and yellowish. Hyperintensity on T2-weighted MRI aids in the identification of neural edema, particularly in instances of neural inflammation without compression, as seen in cases of cubital tunnel syndrome^56^.

The ability to detect inflammation surpasses the capabilities of the ultrasound which preferentially detects neural compressions, making MRI a superior choice in discerning neural inflammation in non-compressive pathologies^56^. While specific hourglass deformities were described in two cases by the operating surgeon, the precise findings of an abrupt change in nerve diameter along with an MRN signal change from hyper-, to hypo-, to hyperintensity characteristic of an hourglass constriction were not seen on an initial MRI evaluation of these two patients^57^. Early branching of the GON and distal rejoining was detected in the MRI of two patients. This splitting is seen on the MRI as a tracked single structured nerve that then forms a fork-like structure and downstream reconnection. In a cadaveric study, only two half-heads out of 40 cadavers demonstrated a division in the GON into two parts, each piercing the trapezius muscle aponeurosis separately and reuniting after passing it^58^. Two patients had the neuropathy of a connection between the GON and the LON. While this connection is described in cadaveric studies at the level of the occiput^58,59^, its visualization on MRI serves as a significant milestone highlighting the MRI’s capability to identify precise anatomical details. In cases where multiple neuropathies are detected in a patient, as illustrated in Figs. 4 and 6, further investigation is warranted to determine whether these neuropathies are interrelated, consequential, or completely independent.

In other neuropathies, such as cubital and carpal tunnel syndromes, electrodiagnostic testing may be utilized to confirm and prognosticate the pathology^60^. However, electrodiagnostic testing is not possible in evaluating the occipital nerve due to its anatomical location. Imaging modalities, specifically MRI, have been explored in other neuropathies. MRI with multiplanar sequences, including high-resolution T1 and heavily T2-weighted fat-suppressed sequences, successfully detected abnormalities of the lumbosacral plexus and lower extremity nerves due to spinal and extraspinal compressions, malignancy, musculoskeletal disease, iatrogenesis, inflammation, and idiopathic diseases^45^. In a study of ulnar neuropathy at the elbow, an axial T2-weighted MRI sequence was evaluated in the detection of compression points associated with ulnar neuropathy. While achieving a perfect correlation in 66% of cases between MRI findings and intra-operative observations, the study also demonstrates a fair-to-moderate level of correlation on Cohen Kappa's measure^61^.

The MRI protocol utilized in our study is distinguished by its ability to concurrently reformat images from the three set sequences simultaneously in any plane, including the oblique plane, without compromising resolution. Additionally, aligning the nerve’s axial plane to be perpendicular to one of the three MRI planes is used to minimize potential morphological swelling artifacts. This combined approach facilitates the seamless tracking of small, tortuous nerves such as the GON.

The aim of this study is to identify and delineate GON-related MRI findings suggestive of compressive neuropathies or pathologies that would be amenable to surgical intervention. These observations were independently recorded intra-operatively and addressed surgically and were later reported upon review of the operative video, thereby validating the retrospective analysis of the MRI scans. While the study delineates MRI's capability to identify morphological features of the GON, it cannot definitively conclude the pathological nature of the observed findings in the current study design based on evaluations of twelve patients.

Despite most patients reporting significant reductions in migraine and headache frequency, duration, and pain severity following surgery, conclusive determination of specific neuropathies resulting from the observed findings post-surgery remains elusive. Future comparative investigations with at least 12 months follow-up between healthy individuals and those with pathology could enhance the understanding of GON-related neuropathies. Establishing a comprehensive database with nerve-tracking maps would significantly augment insights into potential neuropathies affecting the GON. The limited statistical analysis due to the small dataset derived from twelve patients is a study limitation. However, this series demonstrates the viability of utilizing MRI to assess the GON pre-operatively and explore the relationship between anatomical and surgical findings in migraine manifestations.

## Conclusion

MRI can visualize the GON in patients with migraine headaches and occipital pain who are being considered for surgery and can detect anatomical features along the nerve, validated by comparison to intra-operative findings. Just as objective imaging modalities have been demonstrated to improve surgical outcomes in other nerve entrapment disorders such as carpal tunnel syndrome^62^, future research is needed to evaluate the diagnostic ability of high-resolution MRI in detecting migraine and occipital neuralgia-related nerve pathologies, decreasing false negatives and guiding surgical and extracranial interventions.

## Data Availability

The datasets used and/or analyzed during the current study available from the corresponding author on reasonable request.
